# 3,5-Dibromo-2-[2,5-dibut­oxy-4-(3,5-dibromo­thio­phen-2-yl)phen­yl]thio­phene

**DOI:** 10.1107/S1600536811045235

**Published:** 2011-11-05

**Authors:** Chin Hoong Teh, Rusli Daik, Muhammad Mat Salleh, Mohamed Ibrahim Mohamed Tahir, Mohammad B. Kassim

**Affiliations:** a School of Chemical Sciences & Food Technology, Faculty of Science & Technology, Universiti Kebangsaan Malaysia, 43600 Bangi, Selangor, Malaysia; b Institut of Microengineering and Nanoelectronics (IMEN), Universiti Kebangsaan Malaysia, UKM 43600 Bangi, Selangor, Malaysia; c Department of Chemistry, Faculty of Science, Universiti Putra Malaysia, 43400 UPM Serdang, Selangor, Malaysia; d Fuel Cell Institute, Universiti Kebangsaan Malaysia, 43600 Selangor, Malaysia

## Abstract

The title mol­ecule, C_22_H_22_Br_4_O_2_S_2_, is centrosymmetric with an inversion centre located at the centre of the benzene ring. The 3,5-dibromo­thio­phene groups are twisted relative to the benzene ring, making a dihedral angle of 41.43 (9)°.

## Related literature

The title compound belongs to the family of aryl­thio­phenes, compounds frequently used as electroluminescent oligomers to produce polymers for LED applications. For a related structure and background references, see: Promarak & Ruchirawat (2007 ▶); Huang *et al.* (2007 ▶). For related structures, see: Li *et al.* (2008 ▶); Kuriger *et al.* (2008 ▶); Ali *et al.* (2008 ▶).
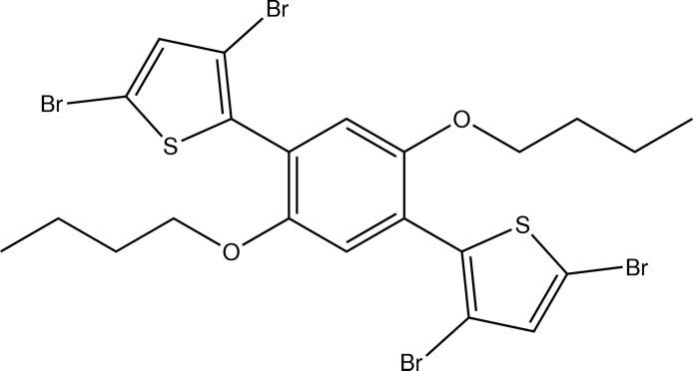


## Experimental

### 

#### Crystal data

C_22_H_22_Br_4_O_2_S_2_
*M* *_r_* = 702.16
Monoclinic, 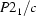

*a* = 13.0156 (3) Å
*b* = 7.8157 (2) Å
*c* = 12.2264 (2) Å
β = 101.027 (2)°
*V* = 1220.78 (5) Å^3^
*Z* = 2
Cu *K*α radiation
μ = 9.79 mm^−1^
*T* = 150 K
0.24 × 0.10 × 0.07 mm

#### Data collection

Oxford Diffraction Gemini diffractometer
Absorption correction: multi-scan (*CrysAlis RED*; Oxford Diffraction, 2006 ▶) *T*_min_ = 0.202, *T*_max_ = 0.547
12067 measured reflections
2349 independent reflections
2272 reflections with *I* > 2σ(*I*)
*R*_int_ = 0.037

#### Refinement

*R*[*F* ^2^ > 2σ(*F* ^2^)] = 0.030
*wR*(*F* ^2^) = 0.084
*S* = 1.10
2349 reflections
138 parameters
H-atom parameters constrained
Δρ_max_ = 0.98 e Å^−3^
Δρ_min_ = −0.54 e Å^−3^

### 

Data collection: *CrysAlis CCD* (Oxford Diffraction, 2006 ▶); cell refinement: *CrysAlis RED* (Oxford Diffraction, 2006 ▶); data reduction: *CrysAlis RED*; program(s) used to solve structure: *SHELXS97* (Sheldrick, 2008 ▶); program(s) used to refine structure: *SHELXL97* (Sheldrick, 2008 ▶); molecular graphics: *SHELXTL* (Sheldrick, 2008 ▶); software used to prepare material for publication: *SHELXTL*, *PLATON* (Spek, 2009 ▶) and *publCIF* (Westrip, 2010 ▶).

## Supplementary Material

Crystal structure: contains datablock(s) I, global. DOI: 10.1107/S1600536811045235/gk2422sup1.cif

Structure factors: contains datablock(s) I. DOI: 10.1107/S1600536811045235/gk2422Isup2.hkl

Supplementary material file. DOI: 10.1107/S1600536811045235/gk2422Isup3.cml

Additional supplementary materials:  crystallographic information; 3D view; checkCIF report

## Figures and Tables

**Fig. 1.**
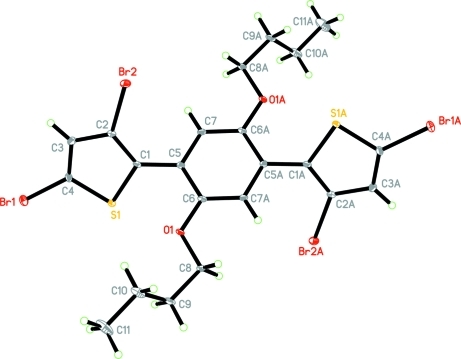
The molecular structure of the title compound, with displacement ellipsoids drawn at the 30% probability level. Symmetry code for atoms with the A label: -x, 1 - y, 1 - z.

**Fig. 2.**
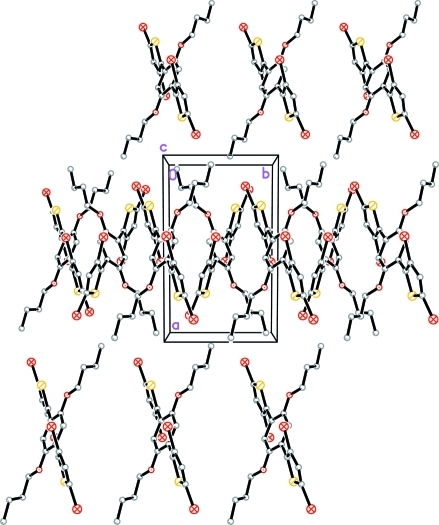
Crystal packing of the title compound viewed down the c-axis.

## supplementary crystallographic information

### Comment

Brominated thiophene-phenylene oligomer with enhanced solubility characteristics due to the presence of alkyloxy substituents such as in the title compound, (I) is an important intermediate to engineer soluble electroluminescent oligomers and polymers for LED applications (Huang *et al.*, 2007).

The structure of I is centrosymmetric with an inversion centre located at the centre of the benzene ring. The mean plane of the central unit [O1/C1/C5/C6/C7/C8/C9/O1A/C1A/C5A/C6A/C7A/C8A/C9A] (A) is approximately planar with the highest deviation of ^±^0.023 (2)° for atoms O1/O1A and the 3,5-dibromothiophene rings are twisted relative to the plane forming a dihedral angle of 41.43 (9)°. Half of the butyloxy groups lie above/below the mean plane A and the mean planes of [C8C9C10C11A] and [C8AC9AC10AC11A] make a dihedral angle of 59.5 (3)° with A. The torsion angle  C8-C9-C10-C11  is 179.7 (3)° and this conformation does not allow for stacking interactions of the aromatic units. Thus quenching of the luminescent effect for polymer generated from this oligomer can be avoided (Fig. 2).

### Experimental

The title compound was prepared according to previously published procedure (Promarak & Ruchirawat, 2007) with a slight modification. *N*-Bromosuccinimide (0.58 g, 3.26 mmol) was added into a solution of 1,4-bis(thiophen-2-yl)-2,5-bis(butyloxy)benzene (0.60 g, 1.55 mmol) in THF:DMF (*v*/*v*=1:1). The mixture was heated under reflux overnight and allowed to cool to ambient temperature prior to addition of water. The compound was extracted into dichloromethane, washed with water and brine solution, dried over anhydrous MgSO_4_ and the solvent was removed by evaporation. Recrystallization of the product from hot dichloromethane solution afforded crystals suitable for single-crystal X-ray diffraction (yield: 63%; m.p. 417-419 K).

### Refinement

The hydrogen positions were calculated geometrically and refined in a riding model approximation with C–H bond lengths in the range 0.93–0.97 Å and *U*_iso_(H) = 1.2*U*_eq_(C) for aromatic and CH_2_ group, and *U*_iso_(H) = 1.5*U*_eq_(C) for methyl group.

### Crystal data

**Table:**

C_22_H_22_Br_4_O_2_S_2_	*F*(000) = 684
*M**_r_* = 702.16	*D*_x_ = 1.910 Mg m^−^^3^
Monoclinic, *P*2_1_/*c*	Melting point = 417–419 K
Hall symbol: -P 2ybc	Cu *K*α radiation, λ = 1.54178 Å
*a* = 13.0156 (3) Å	Cell parameters from 7985 reflections
*b* = 7.8157 (2) Å	θ = 3–71°
*c* = 12.2264 (2) Å	µ = 9.79 mm^−^^1^
β = 101.027 (2)°	*T* = 150 K
*V* = 1220.78 (5) Å^3^	Prismatic, yellow
*Z* = 2	0.24 × 0.10 × 0.07 mm

### Data collection

**Table:**

Oxford Diffraction Gemini diffractometer	2349 independent reflections
Radiation source: fine-focus sealed tube	2272 reflections with *I* > 2σ(*I*)
graphite	*R*_int_ = 0.037
ω/2θ scans	θ_max_ = 71.0°, θ_min_ = 3.5°
Absorption correction: multi-scan (*CrysAlis RED*; Oxford Diffraction, 2006)	*h* = −15→15
*T*_min_ = 0.202, *T*_max_ = 0.547	*k* = −9→9
12067 measured reflections	*l* = −14→14

### Refinement

**Table:**

Refinement on *F*^2^	Secondary atom site location: difference Fourier map
Least-squares matrix: full	Hydrogen site location: inferred from neighbouring sites
*R*[*F*^2^ > 2σ(*F*^2^)] = 0.030	H-atom parameters constrained
*wR*(*F*^2^) = 0.084	*w* = 1/[σ^2^(*F*_o_^2^) + (0.0574*P*)^2^ + 0.8587*P*] where *P* = (*F*_o_^2^ + 2*F*_c_^2^)/3
*S* = 1.10	(Δ/σ)_max_ = 0.001
2349 reflections	Δρ_max_ = 0.98 e Å^−^^3^
138 parameters	Δρ_min_ = −0.54 e Å^−^^3^
0 restraints	Extinction correction: *SHELXL97* (Sheldrick, 2008), Fc^*^=kFc[1+0.001xFc^2^λ^3^/sin(2θ)]^-1/4^
Primary atom site location: structure-invariant direct methods	Extinction coefficient: 0.0057 (3)

### Special details

**Table:**

Experimental. The crystal was placed in the cold stream of an Oxford Cryosystems open-flow nitrogen cryostat (Cosier & Glazer, 1986) with a nominal stability of 0.1 K.(Cosier, J. & Glazer, A.M., 1986. J. Appl. Cryst. 105 107.)
Geometry. All e.s.d.'s (except the e.s.d. in the dihedral angle between two l.s. planes) are estimated using the full covariance matrix. The cell e.s.d.'s are taken into account individually in the estimation of e.s.d.'s in distances, angles and torsion angles; correlations between e.s.d.'s in cell parameters are only used when they are defined by crystal symmetry. An approximate (isotropic) treatment of cell e.s.d.'s is used for estimating e.s.d.'s involving l.s. planes.
Refinement. Refinement of *F*^2^ against ALL reflections. The weighted *R*-factor *wR* and goodness of fit *S* are based on *F*^2^, conventional *R*-factors *R* are based on *F*, with *F* set to zero for negative *F*^2^. The threshold expression of *F*^2^ > σ(*F*^2^) is used only for calculating *R*-factors(gt) *etc*. and is not relevant to the choice of reflections for refinement. *R*-factors based on *F*^2^ are statistically about twice as large as those based on *F*, and *R*- factors based on ALL data will be even larger.

### Fractional atomic coordinates and isotropic or equivalent isotropic displacement parameters (Å^2^)

**Table:**

	*x*	*y*	*z*	*U*_iso_*/*U*_eq_	
Br1	0.85564 (2)	0.22645 (4)	0.97981 (2)	0.02292 (14)	
Br2	0.43160 (2)	0.45620 (4)	0.82956 (2)	0.01904 (14)	
S1	0.74092 (5)	0.36060 (9)	0.74910 (5)	0.01532 (18)	
O1	0.70059 (14)	0.6197 (2)	0.57663 (15)	0.0142 (4)	
C1	0.60951 (19)	0.4183 (3)	0.7216 (2)	0.0119 (5)	
C2	0.56937 (19)	0.3982 (3)	0.8164 (2)	0.0122 (5)	
C3	0.6403 (2)	0.3333 (3)	0.9103 (2)	0.0143 (5)	
H3	0.6239	0.3128	0.9799	0.017*	
C4	0.7353 (2)	0.3055 (3)	0.8837 (2)	0.0143 (5)	
C5	0.5550 (2)	0.4615 (3)	0.6079 (2)	0.0115 (5)	
C6	0.6011 (2)	0.5643 (3)	0.5364 (2)	0.0113 (5)	
C7	0.4545 (2)	0.3963 (3)	0.5690 (2)	0.0125 (5)	
H7	0.4243	0.3250	0.6150	0.015*	
C8	0.7491 (2)	0.7284 (3)	0.5067 (2)	0.0158 (5)	
H8A	0.7077	0.8311	0.4876	0.019*	
H8B	0.7549	0.6692	0.4384	0.019*	
C9	0.8561 (2)	0.7736 (4)	0.5713 (3)	0.0193 (6)	
H9A	0.8917	0.8437	0.5247	0.023*	
H9B	0.8963	0.6692	0.5884	0.023*	
C10	0.8543 (2)	0.8692 (4)	0.6796 (3)	0.0278 (7)	
H10A	0.8147	0.9742	0.6627	0.033*	
H10B	0.8186	0.7995	0.7264	0.033*	
C11	0.9630 (3)	0.9124 (5)	0.7433 (4)	0.0411 (9)	
H11A	1.0016	0.8086	0.7632	0.062*	
H11B	0.9574	0.9744	0.8097	0.062*	
H11C	0.9987	0.9813	0.6974	0.062*	

### Atomic displacement parameters (Å^2^)

**Table:**

	*U*^11^	*U*^22^	*U*^33^	*U*^12^	*U*^13^	*U*^23^
Br1	0.0157 (2)	0.0312 (2)	0.0196 (2)	0.00459 (11)	−0.00230 (13)	0.00655 (11)
Br2	0.01321 (19)	0.0258 (2)	0.0200 (2)	0.00462 (10)	0.00798 (13)	0.00194 (10)
S1	0.0078 (3)	0.0252 (4)	0.0130 (3)	0.0008 (2)	0.0021 (2)	0.0026 (2)
O1	0.0071 (8)	0.0178 (9)	0.0168 (9)	−0.0044 (7)	−0.0001 (7)	0.0041 (7)
C1	0.0075 (11)	0.0121 (11)	0.0160 (13)	−0.0016 (9)	0.0024 (10)	0.0002 (10)
C2	0.0096 (12)	0.0118 (11)	0.0157 (12)	−0.0010 (9)	0.0037 (9)	−0.0015 (10)
C3	0.0155 (13)	0.0152 (13)	0.0130 (12)	0.0008 (10)	0.0045 (10)	0.0012 (10)
C4	0.0131 (13)	0.0151 (12)	0.0133 (12)	0.0009 (10)	−0.0010 (10)	0.0026 (10)
C5	0.0097 (12)	0.0128 (12)	0.0124 (12)	0.0005 (9)	0.0034 (10)	0.0003 (9)
C6	0.0078 (12)	0.0104 (12)	0.0160 (12)	−0.0019 (9)	0.0032 (10)	−0.0024 (9)
C7	0.0099 (12)	0.0130 (12)	0.0157 (12)	−0.0018 (10)	0.0048 (9)	0.0023 (9)
C8	0.0102 (13)	0.0181 (13)	0.0193 (13)	−0.0037 (10)	0.0036 (11)	0.0050 (10)
C9	0.0080 (13)	0.0205 (14)	0.0289 (15)	−0.0028 (10)	0.0025 (11)	0.0040 (11)
C10	0.0136 (15)	0.0281 (16)	0.0406 (19)	−0.0038 (11)	0.0021 (13)	−0.0073 (13)
C11	0.0219 (17)	0.039 (2)	0.057 (2)	−0.0070 (15)	−0.0077 (16)	−0.0178 (18)

### Geometric parameters (Å, °)

**Table:**

Br1—C4	1.874 (3)	C7—C6^i^	1.387 (4)
Br2—C2	1.886 (3)	C7—H7	0.9300
S1—C4	1.716 (3)	C8—C9	1.506 (4)
S1—C1	1.738 (3)	C8—H8A	0.9700
O1—C6	1.365 (3)	C8—H8B	0.9700
O1—C8	1.434 (3)	C9—C10	1.524 (4)
C1—C2	1.368 (4)	C9—H9A	0.9700
C1—C5	1.474 (4)	C9—H9B	0.9700
C2—C3	1.422 (4)	C10—C11	1.517 (4)
C3—C4	1.355 (4)	C10—H10A	0.9700
C3—H3	0.9300	C10—H10B	0.9700
C5—C7	1.400 (4)	C11—H11A	0.9600
C5—C6	1.404 (4)	C11—H11B	0.9600
C6—C7^i^	1.387 (4)	C11—H11C	0.9600
			
C4—S1—C1	91.70 (13)	O1—C8—H8A	110.3
C6—O1—C8	117.94 (19)	C9—C8—H8A	110.3
C2—C1—C5	129.2 (2)	O1—C8—H8B	110.3
C2—C1—S1	109.12 (19)	C9—C8—H8B	110.3
C5—C1—S1	121.31 (19)	H8A—C8—H8B	108.5
C1—C2—C3	115.5 (2)	C8—C9—C10	113.8 (2)
C1—C2—Br2	124.6 (2)	C8—C9—H9A	108.8
C3—C2—Br2	119.89 (19)	C10—C9—H9A	108.8
C4—C3—C2	110.2 (2)	C8—C9—H9B	108.8
C4—C3—H3	124.9	C10—C9—H9B	108.8
C2—C3—H3	124.9	H9A—C9—H9B	107.7
C3—C4—S1	113.4 (2)	C11—C10—C9	112.8 (3)
C3—C4—Br1	126.5 (2)	C11—C10—H10A	109.0
S1—C4—Br1	120.03 (15)	C9—C10—H10A	109.0
C7—C5—C6	118.7 (2)	C11—C10—H10B	109.0
C7—C5—C1	119.1 (2)	C9—C10—H10B	109.0
C6—C5—C1	122.2 (2)	H10A—C10—H10B	107.8
O1—C6—C7^i^	123.7 (2)	C10—C11—H11A	109.5
O1—C6—C5	116.5 (2)	C10—C11—H11B	109.5
C7^i^—C6—C5	119.8 (2)	H11A—C11—H11B	109.5
C6^i^—C7—C5	121.5 (2)	C10—C11—H11C	109.5
C6^i^—C7—H7	119.2	H11A—C11—H11C	109.5
C5—C7—H7	119.2	H11B—C11—H11C	109.5
O1—C8—C9	107.2 (2)		

Symmetry codes: (i) −*x*+1, −*y*+1, −*z*+1.

### Hydrogen-bond geometry (Å, °)

**Table:**

*D*—H···*A*	*D*—H	H···*A*	*D*···*A*	*D*—H···*A*
C7—H7···Br2	0.93	2.80	3.289 (2)	114
